# Microfluidics in Sickle Cell Disease Research: State of the Art and a Perspective Beyond the Flow Problem

**DOI:** 10.3389/fmolb.2020.558982

**Published:** 2021-03-08

**Authors:** Anupam Aich, Yann Lamarre, Daniel Pereira Sacomani, Simone Kashima, Dimas Tadeu Covas, Lucimara Gaziola de la Torre

**Affiliations:** ^1^Intel Corporation, Hillsboro, OR, United States; ^2^Center for Cell-based Therapy, Regional Blood Center of Ribeirão Preto, Ribeirão Preto Medical School, University of São Paulo, Ribeirão Preto, Brazil; ^3^Department of Material and Bioprocess Engineering, School of Chemical Engineering, University of Campinas (UNICAMP), Campinas, Brazil

**Keywords:** sickle microfluidics, sickle cell disease, exosomes, organ-on-chip, drug screening, cell–cell interactions, endothelialized microfluidics, neutrophil-platelet aggregates

## Abstract

Sickle cell disease (SCD) is the monogenic hemoglobinopathy where mutated sickle hemoglobin molecules polymerize to form long fibers under deoxygenated state and deform red blood cells (RBCs) into predominantly sickle form. Sickled RBCs stick to the vascular bed and obstruct blood flow in extreme conditions, leading to acute painful vaso-occlusion crises (VOCs) – the leading cause of mortality in SCD. Being a blood disorder of deformed RBCs, SCD manifests a wide-range of organ-specific clinical complications of life (in addition to chronic pain) such as stroke, acute chest syndrome (ACS) and pulmonary hypertension in the lung, nephropathy, auto-splenectomy, and splenomegaly, hand-foot syndrome, leg ulcer, stress erythropoiesis, osteonecrosis and osteoporosis. The physiological inception for VOC was initially thought to be only a fluid flow problem in microvascular space originated from increased viscosity due to aggregates of sickled RBCs; however, over the last three decades, multiple molecular and cellular mechanisms have been identified that aid the VOC *in vivo*. Activation of adhesion molecules in vascular endothelium and on RBC membranes, activated neutrophils and platelets, increased viscosity of the blood, and fluid physics driving sickled and deformed RBCs to the vascular wall (known as margination of flow) – all of these come together to orchestrate VOC. Microfluidic technology in sickle research was primarily adopted to benefit from mimicking the microvascular network to observe RBC flow under low oxygen conditions as models of VOC. However, over the last decade, microfluidics has evolved as a valuable tool to extract biophysical characteristics of sickle red cells, measure deformability of sickle red cells under simulated oxygen gradient and shear, drug testing, *in vitro* models of intercellular interaction on endothelialized or adhesion molecule-functionalized channels to understand adhesion in sickle microenvironment, characterizing biomechanics and microrheology, biomarker identification, and last but not least, for developing point-of-care diagnostic technologies for low resource setting. Several of these platforms have already demonstrated true potential to be translated from bench to bedside. Emerging microfluidics-based technologies for studying heterotypic cell–cell interactions, organ-on-chip application and drug dosage screening can be employed to sickle research field due to their wide-ranging advantages.

## Introduction

Sickle cell disease (SCD) is a monogenic vascular disorder originating from a single point mutation of beta globin gene of the human hemoglobin, affecting people from African, middle eastern, north Indian and Mediterranean ancestry (Aich et al., 2019). It is estimated that every year about 300,000 children are born with SCD world-wide (Uzunova et al., 2010). At low oxygen concentration, the hemoglobin molecules self-polymerize in long fibers and deform the red blood cells (RBCs) causing more viscous blood flow than normal healthy individual. The very nature of periodic oxygenation-deoxygenation of RBCs leads to recurrent sickling-unsickling of RBCs resulting in persistent presence of sticky RBCs in the blood flow in sickle patients. Vaso-occlusive crises (VOCs) are the major life-threatening acute events of SCD during which the blood flow is obstructed by RBC and other cell aggregates causing severe pain. Other acute complications involve stroke, acute chest syndrome (ACS) and pulmonary hypertension. Persistent inflammation, oxidative stress and activated endothelium facilitate multi-faceted chronic complications involving failure or distress in multiple organs of the body (Ballas et al., 2012; Rees and Gibson, 2012). The disease burden is immense on the quality of life, and chronic pain in adult patients add to the burden from acute complications and end organ damage. While origin of this disease is monogenic, pleotropic effects are present and disease outcomes are clinically variable making the prognosis of the disease unpredictable. Measures to reduce the morbidity and mortality of sickle cell anemia include prophylactic penicillin therapy and substitute in infants and children, and hydroxyurea (HU), the 30-year old FDA approved drug for SCD, in adults (Ataga and Desai, 2018). Very recently, L-glutamine, Crizanlizumab, and Voxelotor have been approved by the US FDA for their efficacy in reducing acute VOC/year in adult population (Ballas, 2020). However, standardization of SCD management plan is difficult as the classification of sickle patients are not straightforward. The only curative treatment of SCD is allogeneic hematopoietic stem cell transplantation, which is expensive and resource-intensive (Aich et al., 2019). Gene therapy approaches under development represent to date potentially definitive cures in SCD, but again price-intensive (Orkin and Bauer, 2019). Therapeutic outcomes are not similar among patients with similar acute or chronic complications and such clinical heterogeneity in the response are yet to be understood from the mechanistic viewpoint. Therefore, elucidating molecular and cellular mechanism of specific complications, identifying novel mediators and pathways and overall addressing patient-specific treatment needs are major areas of improvement in sickle cell research.

**FIGURE 1:**
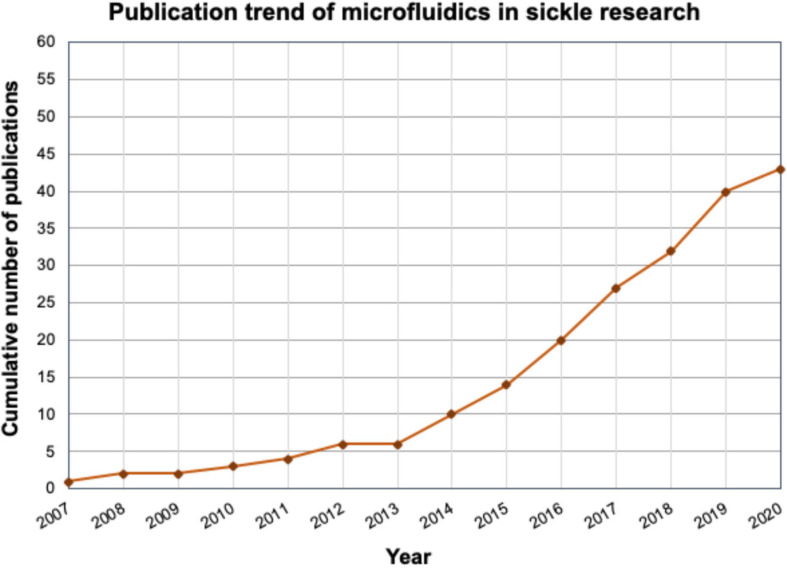
Cumulative trend of publication number of microfluidics-based research in sickle cell field starting at 2007. A rapid progress is observable in sickle microfluidics field. The detailed list is shown in Supplementary Table 1.

In recent decades, the advent of microfluidic platforms has enabled researchers to explore pathophysiological events at the cellular and molecular level in addition of their great utility in diagnostics (Sackmann et al., 2014). Similarly, sickle cell researchers have been involved in utilizing microfluidic technology to understand how VOCs happen during flow of mixture of rigid and non-rigid RBCs, how hypoxia-reoxygenation affects the sickle RBC deformability and their biophysical properties, how microvascular geometry may potentiate occlusion, how non-RBC systemic cells interact with each other and/or vascular endothelium to facilitate endothelial adhesion that aid orchestration of VOC, how blood rheology is distinct for sickle and healthy individuals, how tonic salinity influences sickling of RBCs etc. Additionally, a number of microfluidics-based SCD diagnostic platform have shown promise to be viable to be used in low resource setting with high accuracy and sensitivity (Alapan et al., 2016a; McGann and Hoppe, 2017). The trend of sickle microfluidics research in terms of publication counts can be seen in Figure 1, and a complete list with all the devices and applications can be found in Supplementary Table 1. can be seen in Being more accurate in recapitulating microvasculature than other *in vitro* platforms, microfluidics pose itself as a versatile platform for aiding both mechanistic and translational studies of SCD. Here, we review the current state of the art of microfluidics studies that are driven toward mechanistic understanding as well as studies that have potential for clinical translation. In this review, we have not included discussion about point-of-care diagnostics, as they have discussed in details elsewhere (Alapan et al., 2016a; McGann and Hoppe, 2017; Ilyas et al., 2020). We classify the devices based on their utility in capturing specific biophysical or physiological events underlying sickle pathobiology and discuss exemplary devices, their characteristics and how their usage are filling in knowledge gaps of molecular and physical aspect of the disease. In the end, we envisage development of novel microfluidics platforms for investigating heterotypic cell–cell interactions in the context of vascular-immune and neuro-immune components of the disease that extends beyond the flow problem. Additionally, we discuss the utility of adopting existing organ-on-chip platforms for studying organ-specific sickle complications, and finally, we highlight the advantage of microfluidics as drug assay and dosage screening platform for rapid development of clinically effective therapeutics.

## Current State-Of-The Art of Microfluidics in Sickle Cell Research

Traditionally, acute VOCs were thought to be obstruction only of the capillary blood flow due to aggregation of deformed RBCs originating from the mutated sickle hemoglobin (HbS) polymerization (Manwani and Frenette, 2013). However, in the last two decades, evidences from *in vitro* and *in vivo* studies have demonstrated that the pathophysiology of VOC is complex and the orchestrating events are not limited to merely sickled RBCs aggregating and adhering to endothelium and blocking the blood flow (Kato et al., 2018). A myriad of inflammatory and adhesion activation mechanisms in concert with oxidative stress is inherent to the disease lead to VOC. Sickled RBCs are prone to damage and chronic hemolysis is characteristic feature of SCD. Hemolysis releases hemoglobin and free heme in the intra-vascular space. Extra-cellular hemoglobin consumes nitric oxide, thus, reducing the vascular tone and contributing to the oxidative stress; while free heme adds to the ongoing inflammatory milieu. Deformed RBCs exhibit adhesion molecules on its surface in addition to activation of adhesion molecules (e.g., ICAM-1, VCAM, P-selectin, E-selectin, etc.) on the vascular endothelium. In addition, sickle RBCs and activated endothelium promote sustained pro-inflammatory environment *in vivo*. Non-RBC cells such as platelets, neutrophils and natural killer cells remain activated and contribute to VOC in extreme situation by forming large aggregates with deformed RBCs. Sickle patients exhibit activated neutrophils and platelet-leukocyte aggregates in circulation (Zhang et al., 2016). Murine studies demonstrated that platelet-neutrophil aggregates may be aided by P-selectin (Polanowska-Grabowska et al., 2010), and recent clinical trials targeting P-selectin resulted in positive outcomes in reducing VOC events in sickle patients (Kutlar et al., 2019). Therefore, complex mechanisms underlying VOC extend beyond sickle RBC shape-induced blood flow obstruction and require delineation of molecular and cellular factors/events that involve non-RBC and non-HbS entities.

With the generation of transgenic mouse models of SCD (Berkeley and Townes mice) (Sagi et al., 2018), in the 1990s and early 2000s, the research on VOC relied on observing and quantifying microvascular stasis in dorsal skin venules using intravital microscopy (Kalambur et al., 2004). While *in vivo* studies provide an insight into complex RBC aggregation process or leucocyte rolling that facilitates VOC, the interactions of these cell types with the vascular endothelium depend on multiple factors – which cannot be discerned utilizing these *in vivo* studies. Dissecting roles of RBC deformation, flow shear, distinct cell–cell interactions, adhesion activation, endothelial permeability/dysfunction, immune activation, and converging/diverging vascular bed geometry are essential to fully understand pathophysiology underlying VOC and beyond in SCD in order to develop mechanism-driven targeted therapeutics. Over the last decade, microfluidics have risen to this occasion to enable sickle cell researchers to fabricate and employ simple devices (Horton, 2017) that emulate microvascular dimensions and/or blood cell-endothelial interactions and facilitate *in vitro* experiments to study RBC sickling and VOC events.

### Modeling VOC as RBC Flow Problem in Microvascular Geometry

**FIGURE 2:**
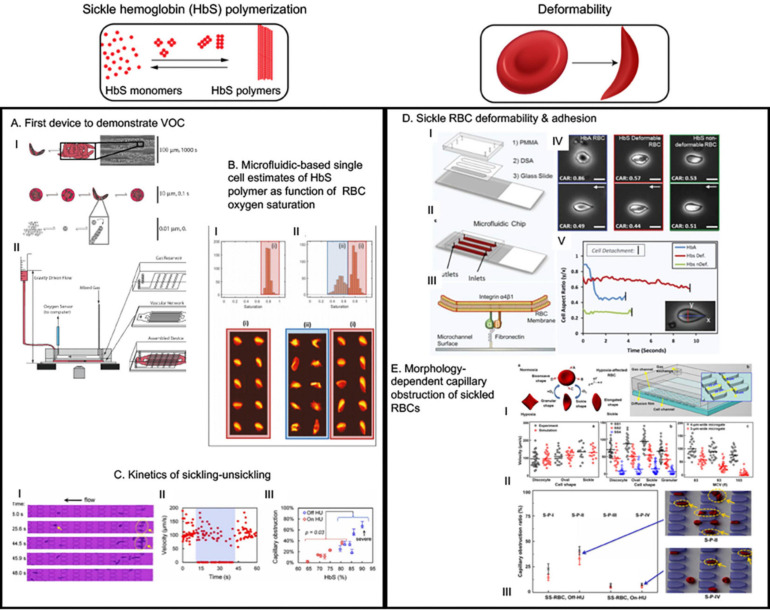
The microfluidic devices in sickle cell research study major molecular and cellular events starting from sickle hemoglobin polymerization and consequent red blood deformability to hemolysis, inflammation, endothelial adhesion and vaso-occlusion (VOC). This figure shows some examples of application of microfluidics in studying HbS polymerization, VOC, and enhanced adhesion of sickled RBCs. **(A)** This was the first device that demonstrated hypoxia induced sickling alone could obstruct microvascular network and demonstrated utility of microfluidics for the study of vaso-occlusion (Horton, 2017). **(B)** The authors utilized high throughput microfluidic single RBC analysis to quantify sickle hemoglobin polymerization in terms of RBC oxygen saturation (Du et al., 2015). **(C)** Transient oxygenation-induced microfluidics-based quantitative single-cell analysis of sickle RBC to understand sickling-unsickling kinetics (Lu et al., 2017). Capillary obstruction percentage correlated with disease severity when hydroxyurea-treated and untreated patient samples were analyzed (III). **(D)** This device incorporated fibronectin-functionalized surface to assay the adhesion characteristics of sickle and normal RBCs (Dominical et al., 2015). They identified that two classes of sickled RBC exist: deformable and non-deformable; and non-deformable sickle RBCs have greater adhesion than deformable sickle RBC. This demonstrated direct correlation of deformation with adhesion (Dominical et al., 2015). **(E)** This study first demonstrated how microfluidics observation of hypoxia-induced morphology variation can help develop simulation-based models to predict experimental biorheological behavior of patient-specific RBCs (Nader et al., 2019). All images presented in this figure have been adapted, modified and/or re-used from original articles as cited. Proper permissions were obtained for the use of the published materials.

Higgins et al. (2007) first demonstrated VOC can be recapitulated in microfluidic devices due to deformed RBCs jamming during flow in channels with dimensions both similar to and greater than the size of RBCs (Figure 2A). An important feature of the device was that the oxygen concentration could be varied using a gas chamber below the fluidic channel with permeable membrane in between. Occlusion initiation under the influence of low oxygen concentration was found to be a slow process (∼124 s), however, dissolution was a rather rapid event (∼22 s). The larger variability in time to jam larger vessels indicates a stochastic process suggesting that only extreme hypoxic conditions may lead to VOC events. While this study was simple in design and did not capitulate the complexities of cellular interactions and adhesions that contribute to VOC, this study demonstrated – for the first time – the feasibility of utilizing microfluidic devices for quantitative study of VOC (Higgins et al., 2007). Subsequently, several microfluidic devices have been developed and implemented to emulate VOC under physiologic oxygen gradients and identify biophysical markers or predictors of VOC (Wood et al., 2012; Du et al., 2015; Lu et al., 2017). Understanding the kinetics of sickling-unsickling under physiologic conditions and consequential effect on microvascular transit time have been a major drive behind these devices. Utilizing one such device, Wood et al. (2012) demonstrated that a rate of change of conductance of blood flow under constant pressure head but with diminishing oxygen concentration is observable and quantifiable for sickle blood while no such change is observed for blood from healthy or sickle trait individuals (Wood et al., 2012). Additionally, sickle blood treated with 5-hydroxymethyl furfural (5-HMF) exhibited significantly reduced rate of change of conductance compared to untreated sickle blood, demonstrating the utility of measurement of such biophysical markers in microfluidic devices to test small molecules that are potential candidates for sickle cell therapeutics (Wood et al., 2012). Additionally, in a modified version of the same device, the same group incorporated microvascular network that contains channels with dimensions of arterial vessels branching into channels resembling post-venule capillaries (Lu et al., 2017). Study using this device with limited sickle blood samples demonstrated that it is possible to have occlusion events at low oxygen tension in the channels upstream of venule-like capillaries. In addition, these results suggest that occlusion events can occur in the timescale of microvascular transit times. Furthermore, similar device was used to estimate sickle Hb polymer content as a function of RBC oxygen saturation collected from high throughput single cell imaging (Figure 2B) (Di Caprio et al., 2019). These observations are crucial for understanding the kinetics of VOC and studying how these events can be interrupted or even slowed down, if not completely stopped from occurring, to facilitate treatment of patients.

With a different approach than channelized flow, Du et al. (2015) investigated sickle RBC flow between micro-pillars with distance among themselves in the size range of RBCs. The motivation was to study the kinetics of sickling and individual RBC deformability under constant pressure and physiologic oxygenation/deoxygenation conditions. Importantly, they defined two parameters to observe sickling kinetics: (a) fraction of RBCs that are sickled at oxygen concentration <5% and (b) delay time (s) required from initiation of deoxygenation to appearance of the first cell taking sickle shape. The RBCs from patients on Hydroxyurea (HU) treatment exhibited significantly longer delay time (>25 s) compared to un-treated patient RBCs, suggesting a beneficial role of HU-treatment in slowing down the sickling process. For combined HU-treated and un-treated group, the sickled RBC fraction under deoxy condition was strongly correlated with the HbS percentage, and capillary obstruction by single RBCs between the micro-pillars exhibited similar correlation (Figure 2C) (Du et al., 2015). Such characterization of sickling-unsickling can potentially serve as aide for prognostic assessments and evaluation of therapeutic benefits in sickle cell clinic. Another study using microfluidic device incorporated much widely separated triangular micro-pillars with acute corners in the flow path of deoxygenated RBCs (Loiseau et al., 2015). While mixture of sickle and normal RBCs did not occlude the channels, the acute corners acted as sites of nucleation and deposition of sickle RBC aggregates. This supports the prominent sites of occlusion in sickle mice during hypoxia/reoxygenation being at venular bifurcations (Kalambur et al., 2004). Healthy RBCs did not produce aggregates. However, in presence of cell-free sickle hemoglobin (HbS), both sickle and healthy RBCs formed aggregates and such aggregation were less prominent in presence of only cell free heathy hemoglobin, thus suggesting a role of cell-free sickle HbS in activation of RBC membrane adhesion (Loiseau et al., 2015). As hemolysis-driven extra-cellular HbS is present in high concentration in sickle patients, free HbS may contribute to VOC via promoting RBC aggregation.

The above devices model VOC as an entrapment of deformed and aggregated RBCs due to a combination of shape change of RBCs and vascular bed geometry. They only offer the kinetics of sickling/unsickling as characteristic and predictive measure of VOC. However, such devices do not take into account the distinct or consorted effects of complex interactions among RBC deformation, flow shear and altered microrheology, sickle RBC-endothelial interactions, and inflammation induced endothelial adhesion-dysfunction which play crucial role in the orchestration of both acute crises and chronic inflammatory-vascular complications in SCD.

### Devices to Study Sickle RBC Deformability, Blood Rheology, and Biomechanical Mechanisms of RBC Aggregation

Red blood cell deformability is the major biomechanical component that drives the micro and macro-rheological properties of blood (Ballas and Mohandas, 2004). Normal RBCs are highly deformable in nature owing to their discoid shape and extra surface area compared to a sphere with same cell volume (Mohandas and Chasis, 1993). Therefore, normal RBCs are able to traverse through capillaries that are of much smaller width than discoid-phase RBC width (∼8 μm). Sickle RBCs are stiff and less deformable than normal red cells. Apart from HbS polymerization under deoxygenation, altered cellular membrane properties (e.g., elasticity and viscosity) and increased cytosolic viscosity due to increased HbS concentration contribute to the reduced RBC deformability (Evans et al., 1984; Ballas and Mohandas, 2004). The HbS polymers exhibit bending moduli of about 1,600 more than the RBC membrane can withstand and four times greater than the outside blood pressure, thus making the deformation of sickle RBCs physiological (Di Caprio et al., 2019). Moreover, cyclic sickling-unsickling causes cell membrane damage. Damaged RBC membrane allows for high permeability of cations upon re-oxygenation and consequent over-hydration lead to increased volume-to-surface area ratio which makes the RBCs further rigid (Barabino et al., 2010). From the fluid mechanics point of view, rheology of whole blood (a highly non-Newtonian fluid) in post-capillary venules is largely influenced by RBC aggregation and deformability (Ballas and Mohandas, 2004). Sickle blood viscosity is higher compared to normal blood and distinct characteristics of sickle blood rheology originates due to the contribution from the heterogeneous mixture of RBC subpopulations in the flow even in the oxygenated condition. In an early effort to characterize rheological behavior of sickle RBC suspensions, Kaul et al. (1983) fractionated sickle RBCs in four sub-populations in terms of their constituent RBC morphology and incremental density: I = least dense reticulocytes, II = discocytes, III = highly dense discocytes, and IV = most dense irreversibly sickled cells (ISCs). Upon deoxygenation, all fractions exhibited similar increase in the levels of viscosity in the bulk viscosity measurements at high shear. However, hemodynamic experiments demonstrate that the peripheral resistance were dramatically increased in the fraction III and IV compared to less dense sub-populations I and II. The significance of this finding lies in the understanding that high and low shear flow may have distinct effects on the RBC aggregation behavior and cumulative resistance achieved in thereafter.

Endothelial adhesion of sickle RBCs is another contributing factor to abnormal blood rheology and blood flow obstruction in SCD. Hoover et al. (1979); Hebbel et al. (1980), and Hebbel (1992) demonstrated *in vitro* that abnormal adhesive interaction of sickle RBCs with the endothelium is present in SCD. Mohandas and Evans (1984) suggested that such adhesion is predominant in the less dense and more deformable RBC population (vs. ISCs), possibly due to greater contact area. This was confirmed by Barabino et al. (1987) by parallel plate flow chamber experiment where the least dense reticulocyte fractions displayed the greatest adhesion to the human endothelial cells. Flowing oxygenated sickle RBCs on a microvascular bed *ex vivo* demonstrated that post-capillary venules are the sites of adhesion of most sickle cells and *in vivo* studies using transgenic sickle mice confirmed similar deposition in post-capillary venules (Kaul et al., 1989b, 1995; Embury et al., 1999). *Ex vivo* observations demonstrated an absence of dense RBC population at sites where there were only adherent cells but no VOC, while at VOC sites a high percentage of dense and ISCs were seen. Based on all these, Kaul et al. (1989a) proposed a 2-step model of VOC. At first, the less dense and deformable sickle cells adhere to the vascular bed making the passage of blood slower. This event promotes hypoxia and consequent jamming of dense and ISCs in the post-capillary venules (which may also have some percentage of deformable adherent cells). However, later it was discovered that leukocyte-mediated adhesion interactions also contribute to the pathogenesis of VOC (Turhan et al., 2002) – discussed later in the review.

Abnormal sickle blood rheology have been studied extensively (Nader et al., 2019). Traditional viscometry, filtration, ektacytometry, micropipette aspiration, atomic force microscopy (AFM), and optical tweezer methods have always been used to measure the rheological properties of blood, RBC deformability, and mechanical and adhesion (with endothelial cells) properties of RBC membranes in isolation from flow (Barabino et al., 2010). However, the earliest example of device mimicking physiological blood flow that quantified the sickle blood rheology was parallel-plate-chamber (Barabino et al., 1987). Recently, sickle researchers have initiated using microfluidics to quantify and characterize both individual cellular biomechanics induced by reduced deformability and collective rheological behavior of sickle RBCs in flow. In an elegant work, Li et al. (2017) characterized individual RBC biomechanics in terms of flow-ability under transient hypoxic conditions in a microfluidic device with capillary channel that contains 15-μm-long, 4-μm-wide, and 5-μm-high micro-pillars as periodic obstacles to the flow (Figure 2E). The RBC samples were density-fractionated in similar fashion as Kaul et al. (1983) did previously. The dense and rigid RBCs (III and IV type) were individually blocked in the microgates during transient hypoxia, but less dense and deformable cells found ways to move forward during the flow via changing direction at the face of obstruction. The traversal velocities of density-fractionated cells were interestingly found to be shape-dependent and more surprisingly, the granular-shaped cells showed less velocity than classical sickle-shaped cells. This unexpected observation was also preserved within the density fractions, thus indicating a within-fraction bio-rheology variability to be present in sickle microenvironment. To test the applicability of this device as a patient-specific prognostic monitoring tool, the authors measured capillary obstruction ratio (= total trapped cells/total cells in channel) for samples from patients with and without hydroxyurea treatment. As expected, HU-treated samples showed significantly less capillary obstruction ratio compared to un-treated samples. All these experiments were also simulated with multi-scale RBC model where RBC membrane elasticity and transient hypoxic conditions were factored in a dissipative particle dynamics model. The simulation results were in excellent agreement with the experimental results, thus establishing this framework as a means of characterizing dynamic behavior of patient-specific individual RBCs under transient hypoxic conditions. Several microfluidic devices studying the bio-rheological properties have been conceived in the last few years, as they are tabulated in Supplementary Table 1.

**FIGURE 3:**
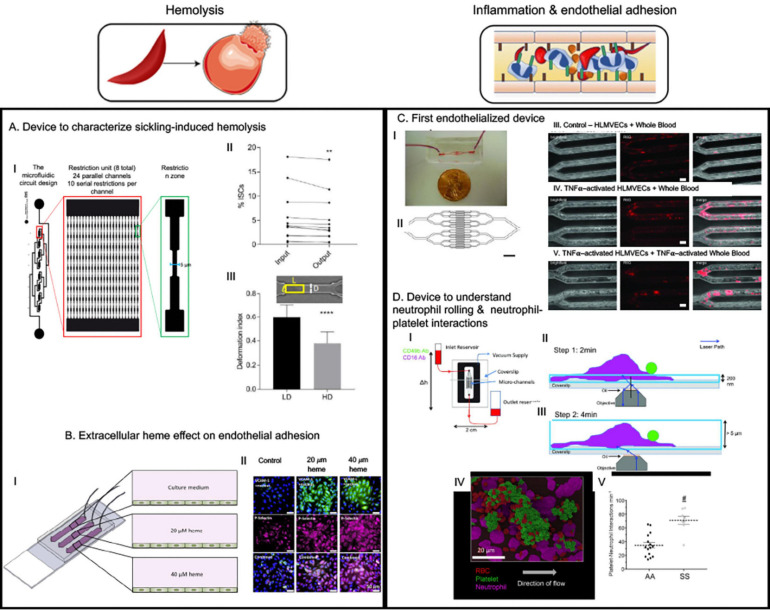
The microfluidic devices in sickle cell research study major molecular and cellular events starting from sickle hemoglobin polymerization and consequent red blood deformability to hemolysis, inflammation, endothelial adhesion and vaso-occlusion (VOC). This figure shows some examples of application of microfluidics in studying hemolysis and inflammation-induced enhanced adhesion of sickled RBCs. **(A)** This device was to demonstrate that less deformable fraction of sickle RBCs are prone to hemolysis when subjected to repeated mechanical stress (Li et al., 2017). **(B)** This device is an endothelialized device where endothelium was activated with free heme simulating hemolysis-driven scenario; higher heme concentration caused more adhesion of RBCs (Mannino et al., 2015). **(C)** “Do-it-yourself” endothelialized device demonstrated that simple off-the-shelf materials can be used to make microfluidic devices to study endothelial adhesion in different geometries relevant to vascular disorders. An important observation was sickle RBCs were more adherent at the bifurcation than in the straight channels and no control RBCs were adhesive at any of the geometry. However, simulating endothelial cells with TNFα did not increase adhesion – indicating a major role of sickled RBCs in adhesion phenomenon (Tsai et al., 2012). **(D)** This device is p-selectin, ICAM-1, and IL-8 -coated to observe neutrophil-rolling and neutrophil-platelet interactions and demonstrated that such interactions are elevated in sickle blood (Embury et al., 2004; Tajima et al., 2009). All images presented in this figure have been adapted, modified and/or reproduced from original articles as cited. All necessary permissions were obtained for the use of the published materials from respective journals.

Another simple microfluidic chip (Lizarralde Iragorri et al., 2018) was recently developed to study the effect of exerting mechanical stress on single sickle red cells (Figure 3A). The device was built with parallel channels that had gradually narrowing walls to squeeze individual cells in flow. The samples were collected at the outlet and then analyzed for quantification of the extra-cellular free hemoglobin as an indicator of RBC lysis under exerted mechanical stress during flow. The results indicated that successive mechanical and increased stress on sickle RBCs can contribute to lysis. A significant observation was that the high-density fraction containing high percentage of ISCs were about 2-fold more susceptible to lysis under such stress compared to low density fraction RBCs from the same sickle blood samples, indicating that less deformable RBCs are more prone to hemolysis under recurrent mechanical stress (Lizarralde Iragorri et al., 2018). Induction of fetal hemoglobin (HbF) can reduce intra-cellular concentration of HbS and thus can reduce sickle RBC sickling (Akinsheye et al., 2011). Analysis of percentage of HbF-positive cells before and after flowing the samples through this microfluidic device indicated the HbF-positive cells were better protected from lysis than HbF-negative cells (Lizarralde Iragorri et al., 2018). More importantly, low-density sickle RBC fractions contained much higher percentage of HbF-positive cells, indicating that higher HbF concentration intra-RBC may restrain RBC density increase, thereby enabling higher deformability and consequently protection from hemolysis. These results demonstrate the utility of simple microfluidic devices to infer deformability and biomechanical properties of sickle red cells to correlate with their rheological properties in relation to factors that govern sickling and consequently contribute to vascular pathology.

### Microfluidics to Study Adhesion: Endothelialized and Protein-Functionalized Microfluidics for Cell–Cell Interactions

Adhesion of RBCs to the vascular endothelium is an essential pre-cursor to facilitating VOC *in vivo*, however, such interactions are not only mediated by expression of adhesion proteins on activated RBCs and endothelial cells, but also through complex cell–cell interactions among RBCs, leukocytes, platelets and endothelium (Manwani and Frenette, 2013). There have been development of two major approaches in modifying the channel beds of microfluidic devices to delineate the role of interactions among sickle RBCs, endothelium, WBCs, platelets and immune cells in the pathobiology of SCD: (1) using endothelialized channels, and (2) using adhesion molecule functionalized channels. We will here describe the general features, utility, outcomes and promises that both of these types of devices hold.

#### Endothelialized Devices

Endothelialization refers to forming a 3-dimensional (3D) endothelial monolayer inside a microchannel to investigate endothelial and other cell interactions mimicking physiologically relevant processes (Figure 3B) (Mannino et al., 2018). The endothelial monolayer is allowed to form after absorption of one of the extracellular matrix (ECM) proteins such as collagen, fibronectin (FN) or laminin (LN) that increases the adhesivity of endothelial cells to the microfluidics device material (Myers et al., 2012). These devices help to recapitulate close to physiological interactions between the systemic RBCs, leukocytes and platelets, and in combination with correct microphysiological dimensions can very closely mimic the capillary or arterial microenvironment (Mannino et al., 2018). SCD is characterized by endothelial adhesion and interactions between endothelium, RBC, leukocytes and platelets, and utilizing endothelialized devices offer the best way to characterize such interactions. Lam lab produced the first endothelialized device with lung microvascular endothelial cell-seeded channels resembling post-capillary venule and arterioles and utilized tumor necrosis alpha (TNFα)-induced endothelial adhesion to demonstrate leukocyte adhesion-induced obstruction/decrease of whole blood flow (Tsai et al., 2011). The rate of blood flow further decreased when the leukocytes were primed with TNFα, thus indicating a role of inflammation in vascular stasis. They used this device to demonstrated that whole blood from patients untreated with HU were able to clog up to 60% of microvascular channels even when fully oxygenated whole blood from HU-treated sickle patients only obstructed up to 4–5% of the network – thus providing evidence that HU-treatment reduces whole blood adhesion or viscosity which possibly provides the treatment benefit (Myers et al., 2012; Tsai et al., 2012). Interestingly, a “do-it-yourself” version of the device with modified channel geometry to add bifurcations showed enhanced RBC aggregation at the bifurcations (Figure 3C) (Mannino et al., 2015), however, TNFα-induced endothelial activation had minimal contribution and sickle RBCs were sufficient to induce such aggregation – demonstrating utility of this device to quantitatively define mediators of VOCs. Another device with much higher endothelialized surface area of 32 mm^2^ (vs. 0.1 mm^2^ in other devices) (Mannino et al., 2015) was used to investigate the adhesion characteristics of sickle RBCs when the endothelial monolayer is pre-treated with micromolar concentrations of heme, as hemolysis-driven intravascular heme is known to activate endothelium and to enhance p-selectin-mediated occlusion. Notable findings from this study were twofold: (1) heme induced adhesion and loss of deformability in a concentration-dependent manner, and (2) when patient samples were clustered based on lactate dehydrogenase (LDH) and reticulocytes – the significantly increased adhesion and decreased deformability in response to heme treatment of endothelial monolayer (also in a concentration-dependent manner) were observed for the patients with high LDH and high reticulocyte counts – indicating a possible hemolysis-dependent clinical and biophysical phenotype segregation among these patients (Kucukal et al., 2018a). Thus, endothelialized microdevices offer more relevant microenvironment to study pathophysiological processes compared to microvascular networks without endothelial monolayers – via simulating characteristic interactions among intravascular cellular entities.

#### Protein-Coated Surface Microfluidics

##### Functionalized with extracellular (ECM) proteins

Commercially available microfluidic devices utilized fibrinogen and collagen functionalized surfaces for cellular adhesion assays, which are being used in sickle research (Proença-Ferreira et al., 2014; Dominical et al., 2015), and similar devices are being developed in academic settings. Plasma FN and endothelial FN are mediators of adhesion between the endothelium and RBCs/neutrophils through the receptor α_4_β_1_. LN is another ECM protein that is also implicated in adhesion process in SCD. Alapan et al. (2014) first demonstrated the utility of FN-treated functionalized microfluidic device to quantitatively describe RBC adhesion and deformability under physiological flow velocities and post-capillary vessel size. Image-based single cell analysis enabled classification of sickle RBCs based on their deformability defined by the aspect ratio change under flow (and also during detachment from adhesive contact) into two classes: deformable and non-deformable. Deformable sickle RBC had significantly lower deformability during flow and at detachment. A field of view analysis of RBCs to find out adherence of RBCs during flow found that the non-deformable sickle RBCs had multiple adhesion sites while deformable sickle RBC had a single adhesion site (Figure 2D) (Alapan et al., 2014). Alapan et al. later improved the algorithm to define the deformability with a parameter dynamic deformability index which is basically the computed rate of change of cell aspect ratio under flow till the time when the cell is detached from the adhesion site. A noteworthy finding was that even at high flow above physiological velocities, there were significantly different non-deformable sickle RBCs compared to deformable sickle RBCs adhered to the surface. This is of significance because while a relationship between RBC deformability and adhesion was always thought to originate from the resistance due to deformed aggregates or the activation of adhesion molecules on endothelium, these data for the first time implicate a direct correlation between these two in a more cause-and-effect manner (Alapan et al., 2016c). Finally, the group implemented SCD biochip, an FN or LN-coated RBC adhesion assay device, to assess over one hundred sickle patient sample and demonstrated that the assay had high sensitivity and accuracy in differentiating hemoglobin phenotypes in addition to finding correlation of clinical parameters such as HbS percentage or LDH with number of adhered cells under different flow conditions and different cell subtypes (non-deformable adherent cells) (Alapan et al., 2016b). Modification of SCD biochip has led to user-friendly characterization of hypoxia-induced (Alapan et al., 2016b) or shear-dependent (Kucukal et al., 2018b) sickle RBC adhesion. In one such study, RBCs from sickle male patients with history of priapism exhibited significantly higher adhesion activity compared to RBC from sickle male patients without history of priapism under hypoxia but not with ambient oxygen concentration (Yuan et al., 2019) – implicating the usefulness of the device in revealing clinical phenotypes or biophysical properties not characterized in traditional non-microfluidic adhesion assays.

Recently, another remarkable study using FN-coated microfluidics device presented – for the first time – quantitative characterization of concurrent adhesion mechanics and HbS polymerization kinetics of sickle RBCs with different deformability under controlled hypoxia (Papageorgiou et al., 2018). The flow shear stress was varied within 0.035–0.085 Pa to allow for sickle RBC adhesion to the FN-functionalized surface of microfluidics device – for the ability to observe the adhesion mechanics in real-time. This microfluidics assay demonstrated that sickle RBCs are more prone to adhesion under hypoxia than normoxia and adhesion propensity is in the following order: highly deformable reticulocytes > deformable discocytes > less deformable discocytes > ISCs. The steps of adhesion dynamics under low shear and hypoxia are as follows:

1. single site adhesion of the sickled cells to FN-coated surface occurs first,
2. the cell flips against single adhesion site to align with the direction of the flow and oscillates with the flow,
3. incremental HbS polymer growth facilitates generation of more adhesion sites via shape change and stops the oscillation of the cell making it permanently adhered.

Further analysis of the discocytes and reticulocyte adhesion phenomena revealed that there is a significant delay between the initial single-site adhesion and final morphological change to sickle shape. The adhesion increases the residence time of the cells under hypoxia which in turn promotes HbS polymerization and consequent shape change-mediated adhesion site increase. Thus, these results demonstrate a mutually synergistic relation between adhesion and HbS polymerization during hypoxia (Papageorgiou et al., 2018). Another important observation is that the most deformable reticulocytes (which are also the most adhesive ones) promote adhesion via HbS polymer-induced protrusions. These outward protrusions to initial membrane/cell boundary causes increase of cell-to-surface contact area thus facilitating enhanced adhesion (vs. mature discocytes and ISCs) possibly due to lower dissociation energy barrier of the lipid bilayer-cytoskeleton of the immature reticulocytes. In an extension of this study, Deng et al. studied the detachment dynamics of the discocytes and ISCs under hypoxia by increasing the shear stress (via changing flow rates of pulsatile flow) up to the point where the single adherent cell detaches from the FN-coated surface (Nader et al., 2019). These critical shear stress required for detachment of single cells represent the adhesion strength and results from the study indicated that the adhesion strength of discocytes were much greater than those of ISCs, thus complementing the prior results of adhesion likeliness being in the same order. Both the studies also incorporated numerical simulations which corroborated the experimental results (Papageorgiou et al., 2018; Deng et al., 2019). Such numerical modeling would be incomprehensible without the leverage of microfluidics to extract certain critical parameters.

Carden et al. (2017) recently utilized both non-endothelialized hypoxia-enabled and endothelialized devices to investigate the effect of infusing intravenous fluids (IVFs) with different tonicities. This approach enabled them to separately determine the effect of IVFs with different tonicity on deformability under normoxic and hypoxic conditions in the former device and also estimate the effect on adhesion using the endothelialized device. This study highlights the relevant advantages of different types of microphysiological devices in the context of decoupling adhesion mechanics and studying deformability. More importantly, they were able to extract an optimum tonicity from these measurements which balances the biophysical changes of RBC and adhesion characteristics, thus indicating translational potential for such approach (Carden et al., 2017).

##### Adhesion and chemoattractant protein-coated surfaces

Adhesion interaction in SCD is not limited to endothelium and blood cells, rather RBC, platelets and neutrophils also mediate intercellular interactions between themselves to form aggregates in flow (Telen, 2014). Such interactions are mediated primarily by P-selectin that is expressed on endothelial cells and platelets in response to inflammation in SCD (Embury et al., 2004; Polanowska-Grabowska et al., 2010). Neutrophil-platelet-RBC aggregates have been demonstrated to be involved in ACS – a form of acute lung injury in the event of VOC in the lung vasculature (Bennewitz et al., 2017), and Sundd lab utilized a unique microfluidic device functionalized with immobilized p-selectin, intercellular adhesion molecule 1 (ICAM-1) and interleukin 8 (IL-8) (Bennewitz et al., 2014, 2017; Jimenez et al., 2017; Vats et al., 2019) to delineate aspects of neutrophil-platelet interactions in forming such aggregates. ICAM-1 is a major adhesion protein expressed on endothelium responsible for leucocyte-endothelial interaction (Kotteas et al., 2014) and IL-8 is a chemoattractant for the neutrophils (Tajima et al., 2009). Thus, this approach captures the major endothelial interactions with neutrophil and platelet without having the need to using endothelialization approach and provides a better visibility toward specific interactions – as has been demonstrated by the initial study (Figure 3D) (Jimenez et al., 2015). Using this approach, the group first demonstrated that P-selectin-mediated neutrophil-platelet interaction is a feature of lung VOC (Bennewitz et al., 2017) and recently, they demonstrated that platelet-derived exosomes that carry IL-1β and caspase-1 can contribute to neutrophil-platelet aggregation, consequently causing lung VOC *in vivo* (Vats et al., 2019). A commercially available E-selectin/ICAM-1-coated microdevice with gradually decreasing width demonstrated that sickle neutrophils were more adherent and occlusive than healthy neutrophils. In the same study sickle RBCs alone were not sufficient to obstruct LN-coated channels and such obstruction was only facilitated by addition of sickle neutrophils to the sickle RBC solution (Dominical et al., 2015). This approach of chemoattractant and adhesive protein functionalized microfluidics, thus, have been successful in validating mediators of VOC *in vitro* and to reveal detailed cell–cell interactions underlying inflammation and its contribution to occlusive adhesion in SCD.

A unique microfluidics-based well-plate platform (White et al., 2015) that utilizes vascular cell adhesion molecule 1 (VCAM-1) for functionalization of the well-plate and pulsatile flow for the assay demonstrated significant adhesion variation in adhesion characteristics of adherent RBCs during pulsatile flow compared to continuous flow. As physiologically blood is pumped in a pulsatile manner, such approaches could enable more appropriate quantification of adhesion characteristics of sickle RBCs. Indeed, this approach proved to be useful in observing effects of novel drugs to reduce sickle RBC adhesion (White et al., 2016; Lancelot et al., 2017).

## Current Challenges and Promising Microfluidics to Better Understand SCD: A Perspective From Addressing Complex Biology

While there has been tremendous progress in the field of sickle cell microfluidics during recent years, majority of the studies focus on understanding or evaluating the adhesion mechanics and biomechanics of vaso-occlusion, in addition to vascular cell–cell interactions. Some technologies such as adhesion protein-coated adhesion assay chips are commercially available and there are devices already developed by several engineering groups to tackle such problems. However, it is still a challenge use microfluidic technology to mimic complex biological systems or problems such as SCD. Consequentially, the usage of complex vasculature systems or organ-on-chip systems have not evident in sickle research field. The microenvironment for mimicking *in vivo* conditions requires elaborated engineering solutions. Moreover, protocols for automated quantification, involving a huge amount of data acquisition requires the development of software and data processing. In this sense, these systems are still under development and it is not a plug-and-play technology for biologists. Complex microdevices require experience major experience in developing them – which is probably why there have been only about 44 articles published over more than a decade of sickle cell microfluidics research. Hence, it is understandable that many of microfluidic technologies that have been availed at different disease states, they have not been yet introduced in sickle cell field.

Additionally, many different microdevices with complexity relevant to sickle microenvironment have been used in other disease states, the lack of standardization of outcomes that is relevant to SCD is absent. For example, while concentration gradient microfluidics have been in use for some time, the absence of standardized characteristics of assessing the outcomes of vascular adhesion or other sickle microenvironmental parameters have made such technologies inapplicable so far. Another major hindrance of progress in sickle microfluidics research has been of less involvement of engineers in sickle research field. While in recent years, few engineers have been involved in fundamentally addressing sickle research using microfluidics technology, the long-standing disconnect of the sickle researchers from physician and biologist community with the engineers have yet to be dissolved. With increasing interaction and collaboration between engineers, biologists and clinicians, sickle research field is currently observing a greater progress toward standardizing some adhesion assays. A characteristic feature of SCD is its heterogeneity and clinical variability, which requires any microfluidic technologies to be validated with hundreds of patient samples – which are not easy to obtain. These limitations pose intrinsic obstacles in the rapid development, standardization and ubiquitous implementation of novel microfluidic devices that are possibly already in use in other research fields.

There are critical needs of devices that can recapitulate the sickle microenvironment not only in terms of vascular endothelial beds but also the interaction with non-vascular heterotypic cells such as neuronal or tissue-resident immune cells. Additionally, it is important to capture the complexity of organ systems that are affected by sickle red cells and inflammatory hemolytic microenvironment. Such interactions can only be recapitulated in microfluidics-based organ-on-chip systems – which could be useful to screen drug compounds and their effects on end-organ damages. Such devices have not been so far utilized or incorporated in SCD research field. Additionally, no standardized platforms exist to screen drug compounds for their efficacy. There are multiple research groups are studying small molecule effects on endothelialized system – however, the translatability of such devices into screening different concentrations of drug molecules or synergistic effectiveness of multiple drugs are still absent. Owing to the versatility of microfluidics, current advancement in modular composite fluidic systems, and most importantly, reflecting on the complexity of sickle microenvironment, organ-specific sickle complications and high patient-specific variability in clinical prognosis, we anticipate and emphasize the need for novel and emerging areas of microfluidics-based technology development for: (1) studying exosome-mediated interactions between endothelium and non-systemic cell immune cells, (2) organ-on-chip platform, and (3) parallelized therapeutic testing platforms – all in the context of sickle research.

### Need for Novel Microfluidics for Studying Exosome-Mediated Vascular-Immune Interactions in Sickle Microenvironment

Distal homo- and heterotypic cell–cell interactions are often mediated via exosomes (Patil et al., 2019), and recently circulating exosomes have garnered attention as a mediator of sickle pathobiology such as endothelial dysfunction (Khalyfa et al., 2016; Lapping-Carr et al., 2017). Exosomes are extracellular nanovesicles released by numerous cell types into biological fluids and known to be involved regulatory physiological as well as in pathophysiological processes, modifying the functional phenotype of target cells (Patil et al., 2019). While circulating microparticles have been investigated in SCD due to their relevance (depending on source) vascular injuries, reticulocytosis, endothelial activation, endothelial adhesion and platelet activation i.e., the major features of SCD (Hebbel and Key, 2016), only very recently, micro-RNAs from plasma-derived exosomes from a set of children with diverse set of SCD-induced complications have been shown to display a molecular signature reflective of disease severity (Khalyfa et al., 2016). Subsequent analysis of exosomes from sickle children with ACS+ and without ACS- demonstrated that the exosomes from ACS + children could alter endothelial integrity and had induced eNOS expression in these cells, more than exosomes from ACS-children (Lapping-Carr et al., 2017). The endothelial permeability measurements were done with electrical cell impedance sensors (ECIS). While ECIS is an appropriate tool for such measurements, direct observation of exosome uptake by endothelial cells was absent in this study (Lapping-Carr et al., 2017). Also, in general, how non-systemic and endothelial cells interact in sickle microenvironment is unknown. SCD being a vascular disease with complex vascular-immune and neuro-immune interactions (Aich et al., 2019), it is essential to understand the complex interplay mechanistically for developing targeted therapeutics. For example, mast cell activation contributes to sickle pathobiology (Vincent et al., 2013) and vascular dysfunction (Tran et al., 2019). However, how mast cells and endothelial cells interact in sickle microenvironment is unknown. Mast cell-derived exosomes have been shown to activate endothelial cells to secrete plasminogen activator inhibitor-1 (PAI-1) (Khalid et al., 2005). Elevated levels of PAI-1 have been found in steady state SCD patients (Hagger et al., 1995) which further increases during VOCs (Nsiri et al., 1997). Therefore, complex heterotypic cell–cell interaction networks via exosomes is conceivable in the scope of SCD, investigation of which is warranted. Microfluidics-based approach can help to decipher molecular mechanisms of exosome-mediated intercellular interactions in sickle microenvironment with an added advantage of direct observation of kinetics of the process – due to ease of being able to membrane-separated chambers for co-culture setups. Thus, microfluidic devices that contain engineered cells that produce exosomes in one chamber and another chamber containing cells that uptakes the transferred exosomes through a permeable membrane that separates the two chambers may help to delineate role of exosomes in modulating endothelial dysfunction and/or other heterotypic inter-cellular interactions in the context of SCD. Additionally, such cell–cell interaction device also offers modeling opportunities for studying blood flow-induced endothelial exosome-secretion and its consequential effects on surrounding microenvironment. We envision that this type of devices to study heterotypic cell–cell interactions and their modifications to simulate sickle microenvironment will offer exciting and innovative approaches to elucidate novel molecular and cellular processes and mediators in SCD.

### Organ-on-Chip Devices to Study Organ-Specific Complications in SCD

**FIGURE 4:**
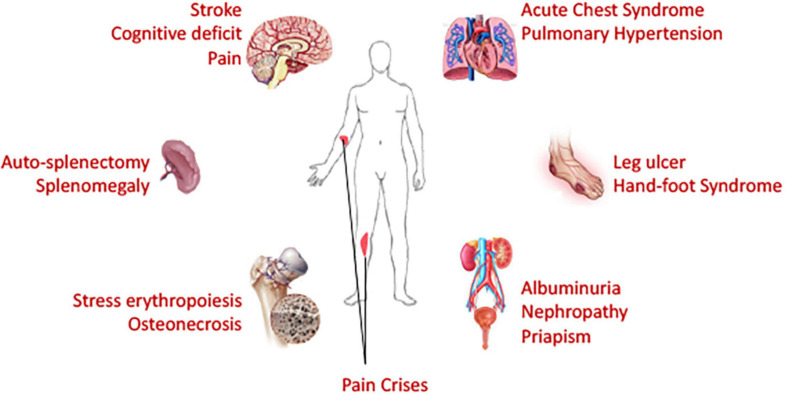
Necessity of organ-on-chip devices: Being a blood disorder with hyperinflammatory and hypercoagulative state, sickle cell disease affects many organs of the body. Chronic and ongoing insults and injuries result in end organ damages. Life threatening acute complications affect brain, lung and spleen; while chronic deleterious effects are observed in many organs. Organ-on-chip devices, if utilized, can provide a holistic view on the macroscopic sickle microenvironment and should facilitate evaluation of efficacy of therapeutic interventions in organ-specific manner. Patient-specific modeling can also be performed via development of organ-on-chip systems from patient-derived stem cells, thus paving the path toward personalized medicine.

Acute VOC pain crisis is the major feature of SCD, and all currently FDA-approved drugs were approved based on the outcome of reduction of VOC events. However, despite organ-specific complications such as stroke in brain, ACS and pulmonary hypertension in the lung, splenomegaly and auto-splenectomy, kidney-renal complications, leg ulcers etc. contributing to life-threatening and life-disabling aspects of SCD – independent of VOC (Figure 4), such complication-specific sickle therapeutics is unavailable due to limited understanding of underlying mechanisms. While current microfluidics devices used in sickle research field can correlate RBC deformation and blood cell-endothelial adhesion well with some clinical parameters to some degree, none captures the organ-specific complex tissue-vasculature interactions and are, therefore, not suitable for studies of organ-specific complications. Integrated microsystems composed of various cell types and extra-cellular matrix and designed with capacity to mimic mechanical, biochemical and functional properties similar to *in vivo* microenvironment are known as organ-on-chip platforms. Organ-on-chip platforms are complex microfluidic systems that feature the physiologically relevant complexity of human biology and can provide more intricate interactions from integration of tissue matrix, vasculature, muscles and other organ-specific cells/tissues in addition to off-chip/on-chip perfusion systems and/or micromachined self-actuating or electro-activated mechanical valves/filtration systems (Zhang B. et al., 2018). Organ-on-chip devices can provide more holistic idea of effects originating from a drug or interventional treatment.

While transgenic sickle mouse models are available that express sickle human hemoglobin (with transgenic control mouse expressing normal human hemoglobin) which recapitulate many features of SCD such as RBC sickling, RBC-leukocyte aggregated-mediated vaso-occlusion, reticulocytosis, hemolysis, inflammation, and acute/chronic pain (Sagi et al., 2018). However, rodent models do not always capture the variety of organ-specific complications. At the same time many drugs that are effective in mouse models, they do not translate into effective therapeutics once tried in humans (Justice and Dhillon, 2016). Additionally, *in vitro* studies with single/two-cell systems do not capture the complexities of human tissue interactions with systemic environment, immune cell responses and other molecular/cellular mediators of complex microenvironment. For example, Rivipansel, a pan-selectin antagonist (selectins are mediators of endothelial adhesion in SCD), demonstrated efficacy in transgenic sickle mouse and endothelial adhesion assay;(Chang et al., 2010) however, the drug failed to demonstrate efficacy in phase III clinical trial in reducing acute VOC crisis events and required opioid usage in sickle children of age 6–11 years (Morris et al., 2013; Telen et al., 2015; Ballas, 2020). While resources are often scarce and disparate for sickle cell research (Lee et al., 2019), such loss of resources in terms of millions of dollars and time of scientists in a path of ineffective drug development adds to the burden of managing this disease. Organ-on-chip platforms may offer faster and less resource-intensive outcome measures of physiologic relevance before the preclinical studies. Since the first lung-on-chip system in 2010 (Huh et al., 2010), major improvements have been made in the field of tissue engineering and microtechnology to build different organ systems such as brain (Bang et al., 2019), lung (Nawroth et al., 2019), kidney (Hagger et al., 1995), spleen (Rigat-Brugarolas et al., 2014; Picot et al., 2015), neural systems (Huval et al., 2015; Pamies et al., 2017; Sharma et al., 2019), and skin (Zhang Q. et al., 2018) among many. The emergence of 3D bioprinting have also been conducive to rapid development of organ-on-chip systems (Miri et al., 2019).

Another aspect of utility of organ-on-chip platforms is addressing the discrepancy and dissimilarities in the form of pathophysiological, genetic and translational landscape for a specific biological event. For example, SCD is a hemolytic anemia and consequent stress erythropoiesis is a major feature of the disease (Zivot et al., 2018). However, erythroid systems respond differently at the molecular and cellular levels in human and mouse (Telen, 2014). While understanding the differences may offer some relief in studying the stress erythropoiesis in transgenic mouse, the inaccessibility of bone marrow coupled with the human/mouse differences makes it difficult to study such processes in real-time. Bone-marrow-on-chip devices using ECM and myeloid cells in concert with vascularized channels (Chou et al., 2020) or human hematopoietic stem cell derived complex 3D microsystems resembling bone-marrow microenvironment (Sieber et al., 2018) may offer insights into mechanisms of the erythropoiesis dysfunction and therapeutic benefits of transfusion or drugs on easing the burden on the hematopoietic niche in SCD. Additionally, such devices will offer benefits to prognostic observation of disease progression at the organ level, which studies are very difficult to perform in humans or mice. Similar on-chip platforms of brain, lung, spleen, skin, peripheral and central nervous system, and kidney with vascularized system are suitable for studies at the microphysiological levels to understand organ-specific comorbidities and disabilities in SCD, such as stroke, ACS, splenomegaly, leg ulcer, pain, renal complications and many more. These devices also enable testing of drug safety, toxicity, efficacy, and relevant biomarker validation in a tissue/organ-specific manner and can provide insights into effects of mono/combination therapeutics (Nawroth et al., 2019). Additionally, organ-on-chip platforms made from patient-specific stem cells can provide insights into the clinical variability and patient-specific therapeutic response, thus, offering a more precision and personalized medicine approach for sickle patients. Finally, body-on-chip devices (Sung et al., 2019) can help with understanding how sickle drugs targeted to reducing HbS polymerization or endothelial adhesion might effect differently at organ-specific level and can provide insights to design combination therapeutics to enhance the treatment outcome (Herland et al., 2020; Novak et al., 2020).

### Emerging Concentration-Gradient Microfluidics for Testing Novel Drugs and Dosage Comparison

Novel drug development requires high-throughput screening of the compounds for their efficacy and also once identified, dosage and concentration identification is a major challenge. Dosage evaluations primarily depend on pre-clinical mouse studies or phase I clinical trials – which is time and resource-intensive. The estimated cost to develop a clinically approved drug is 2.5 billion dollars (DiMasi et al., 2016). However, two thirds of the total costs for drug development are spent in the initial research phases (Paul et al., 2010). Also, we have discussed that development of Rivipansel, an anti-adhesion molecule, for a decade as sickle cell drug failed in the phase III clinical trials. Despite renewed interest at the national/international policy level and among pharmaceutical industries for developing sickle drugs, the long history of disparate funding for sickle research (Lee et al., 2019) has limited the identification of drug targets and their subsequent development. Thus, it is imperative to develop novel strategies to speed up and decrease costs for drug screening by eliminating drug candidates as early as possible in the approval process is beneficial, since to fail early is to fail cheaply (Zhang and Radisic, 2017). Microfluidics offers high throughput screening of drug compounds owing to ease of observing cellular behavior, compartmentalization, parallelization and generating concentration gradients. Concentration gradients have been investigated in different microchips applications and since different conditions can be investigated at the same experiment including replicates, resulting in high throughput screening options (Atencia et al., 2009; Cimetta et al., 2010; Wang et al., 2017; Regnault et al., 2018). These microchips are designed to manipulate fluids in a way where the reagent concentration is known in space and time.

**FIGURE 5:**
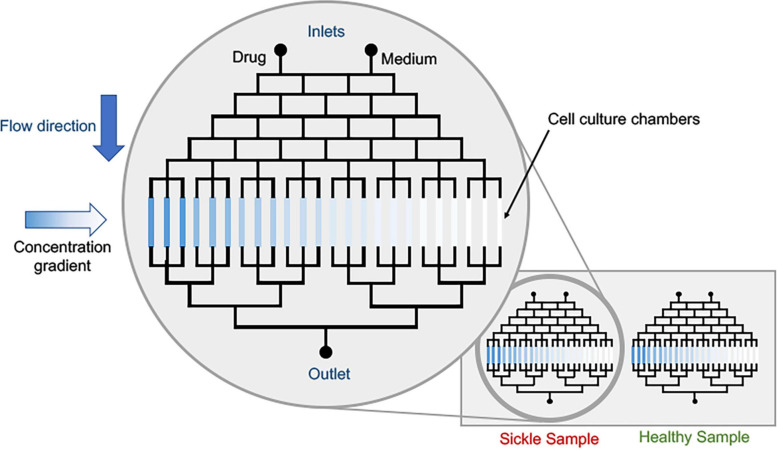
Conceptual schematic of drug dosage screening device. The cell culture platforms can be the current state of sickle microfluidic devices that can measure deformability, adhesion or other characteristic parameters pertaining to sickle pathobiology. Parallelization of healthy and sickle samples is possible via multiplexing.

Different concentration gradients can be achieved in different mixing systems: convection and diffusion-based (Oliveira et al., 2016; Regnault et al., 2018; Vit et al., 2019). Convection-based concentration gradients are formed via laminar flow mixing. In this case, the contact between two streams with different concentrations promotes solute mixing and depending on the geometry, a linear concentration gradient can be generated, and diffusion-based concentration gradients have no net flow and mass transport occurs only via diffusion (Regnault et al., 2018). These different strategies to generate concentration gradients, that can vary from linear to exponential ones (Wang et al., 2017) and the design can be selected according to the research purpose. In sickle research context, the use of already developed microfluidic techniques to investigate VOC, RBC adhesion, inflammation and/or blood cell-endothelium interactions coupled with approaches to generate concentration gradients to evaluate dosage values of a single drug; a conceptual device is shown in Figure 5. Multiple devices have demonstrated utility in evaluating novel drug effects on HbS polymerization, RBC sickling, RBC membrane damage, microrheology and endothelial adhesion (Du et al., 2015; Lu et al., 2019; Hansen et al., 2020; Man et al., 2020; Noomuna et al., 2020). Moreover, often sickle patients require combination therapeutics. Thus, evaluation of synergistic effect of the combination of two drugs can also be explored, in order to minimize drug administration. Microfluidic tests can be used prior to *in vivo* studies, minimizing animal tests and speeding up the results. Finally, pre-mixing and generating gradients before administering drugs into organ-on-chip devices can provide approaches to evaluate effects on multi-cellular and tissue levels. We propose these approaches be evaluated not only in academic setting, but also for industrial level drug combination evaluation – as we see a surge in the pharmaceutical companies’ interest in developing sickle drug (Ataga and Desai, 2018).

## Conclusion

Sickle cell research field has seen rise in the microfluidics technologies for over a decade now, while organ-on-chip systems in other fields have become viable options to mimic micro- and macro-physiological processes of human biology. A critical understanding of how the future of sickle cell microfluidics research will look like lies at the intersection of three major perspective. Firstly, the decades-old *in vitro* studies performed in cell culture petri dish to investigate basic properties of sickle red cells such as deformability, adhesion and microrheology are now being improved by incorporation of vascular components in microfabricated systems. While we acknowledge that the current state of art of sickle microfluidics have provided us basic devices to study these properties in more physiologically relevant approach that were inconceivable before, we must recognize the need for more complex systems such as heterotypic cell–cell interaction devices and organ-on-chip platforms in enhancing mechanistic understanding of the disease process. Secondly, currently there is limited understanding of the relevant critical components that truly represents suitable or quasi-suitable configuration for specific tissue or vascular architecture and/or combination of cellularized modules in microphysiological and organ-on-chip systems. Therefore, it is imperative that standardization of fabrication processes, validation methods, characterization of relevant molecular and cellular mediators and physiologically relevant input and output measures needs to be assessed in a more rational manner to establish guidelines that distinguishes good and bad design – with relevance to SCD physiology and by the consensus among the clinicians and researchers in the field. Finally, the future of sickle microfluidics is not solely limited to the development of the device itself, but also requires development of novel imaging, measurement and analytics approaches, in addition to rapidly testing the utility of such devices in a more clinically oriented manner. However, accomplishment of such advancements would require the inter-disciplinary approach from tissue/microfabrication engineers, biologists, hematologists, data acquisition/analytics specialists, imaging scientists, polymer scientists and many other scientific and technological professionals.

The promise of microfluidics in sickle research is multifaceted, however, microfabrication facilities and development of organ-on-chip systems are resource-intensive. Therefore, such endeavors must be facilitated at the national policy-level by ensuring that more funding is available for innovation in countries like Brazil and low/middle-income-countries in Africa where unmet need of disease management for sickle patients is prevalent. Currently the scientific community in the developed countries is racing to find a gene-therapy-based cure for SCD, however, to make such cures affordable for everyone in the long run is quite a challenge. In the meantime, microfluidics can help us to understand novel mechanisms, identify new drug targets, screen novel drug molecules and finally model the drug delivery and safety/efficacy in organ-on-chip platforms. And we should pursue these promising avenues with clear scientific and therapeutic goals to help ease the burden of sickle patients.

## Author Contributions

AA, LGT, and YL conceived and designed the research, performed literature search, and wrote and edited the manuscript. AA, YL, and DS prepared the figures. DS performed the literature search, wrote and edited manuscript, and prepared Supplementary Table 1. SK and DC supervised YL. All authors contributed to the article and approved the submitted version.

## Conflict of Interest

AA was employed by Intel Corporation and declares that no grant/salary support was provided by Intel Corporation for this work. The remaining authors declare that the research was conducted in the absence of any commercial or financial relationships that could be construed as a potential conflict of interest.
